# Analysis of the association between testosterone and cardiovascular disease potential risk factor apolipoprotein B in adult males without cancer: national health and nutrition examination survey 2011-2016

**DOI:** 10.3389/fendo.2024.1304344

**Published:** 2024-02-16

**Authors:** Zhiyi Chen, Enpu Zhang, Lu Gan, Ganggang Jiang, Qilin Duan, Mou Huang, Huizhen Li, Guixiao Huang

**Affiliations:** ^1^ Department of Urology, The Third Affiliated Hospital of Shenzhen University, Shenzhen University School of Medicine, Shenzhen, China; ^2^ Department of Urology, The Third Affiliated Hospital of Shenzhen University, Shenzhen Luohu Hospital Group Luohu People’s Hospital, Shenzhen, China

**Keywords:** testosterone, ApoB, LDL-C, CVD, NHANES

## Abstract

**Background:**

Over the years, there has been extensive exploration of the association between testosterone and lipid profiles, yet the precise mechanisms underlying their interaction remain incompletely elucidated. Similarly, there is a dearth of research on the correlation between serum apolipoprotein B (apoB) and serum total testosterone (TT), particularly within specific populations.

**Methods:**

We conducted a cross-sectional study to assess the relationship between serum TT concentration and serum apoB concentration. Using the National Health and Nutrition Examination Survey (NHANES) from 2011 to 2016, we employed weighted generalized linear models, weighted univariate, weighted multivariate analysis, and smooth curve fitting to assist in exploring the relationship between serum TT and apoB. Serum apoB concentration served as the independent variable, and serum TT concentration as the dependent variable. ApoB was divided into four quartiles—Q1 (<0.7g/L, N=691), Q2 (≥0.7g/L to <0.9g/L, N=710), Q3 (≥0.9g/L to <1.1g/L, N=696), and Q4 (≥1.1g/L, N=708)—thereby further solidifying the stable association between the two. Additionally, the application of smooth curve fitting will contribute to a more detailed elucidation of the specific relationship between serum TT concentration and serum apoB concentration under different factors (Drinking, Smoke, Diabetes, Hypertension, and High cholesterol level.).

**Results:**

The results indicate a negative correlation between serum TT concentration and apoB concentration (β=-113.4; 95% CI: -146.6, -80.2; P<0.001). After adjusting for confounding variables, the negative correlation between apoB concentration and TT concentration remains significant (β=-61.0; 95% CI: -116.7, -5.2; P=0.040). When apoB concentration was converted from a continuous variable to a categorical variable (quartiles: Q1<0.7g/L; Q2:≥0.7g/L to<0.9g/L; Q3:≥0.9g/L to <1.1g/L; Q4: ≥1.1g/L), TT level of participants in the highest quartile (≥1.1g/L) was -47.2 pg/mL (95% CI: -91.2, -3.3; P=0.045) lower than that in the lowest quartile (<0.7g/L). The smooth curve fitting diagram revealed differences in the relationship between TT concentration and apoB among individuals with different cardiovascular disease (CVD) risk factors.

**Conclusions:**

This study elucidates a robust inverse correlation between serum TT concentration and apoB concentration, maintaining statistical significance even upon adjustment for confounding factors. These findings present a promising avenue for addressing the prevention and treatment of low testosterone and CVD.

## Introduction

Testosterone is produced by Leydig cells within the testis, playing a crucial role in the differentiation of the male reproductive tract and masculinization of the external genitalia during fetal development (1). The release of testosterone is regulated via the hypothalamus-pituitary-testis interstitial cell axis (2). Decreased levels of serum testosterone (at or below 300 ng/dL) often present with diminished sexual thoughts and frequency, weight gain, and erectile dysfunction (3, 4). Gonadotropin-releasing hormone (GnRH) binds to membrane receptors on the pituitary gonadotropes, stimulating the biosynthesis and secretion of luteinizing hormone (LH) (5, 6). LH binds to LHR on the surface of interstitial cells, initiating intracellular signal transduction. Dufau and Catt (7) demonstrated that cAMP is generated in response to LH. The cAMP pathway through protein kinase A (PKA) is crucial in steroidogenesis. Furthermore, the chronic stimulation of interstitial cells by LH and cAMP is vital for regulating the expression levels of proteins and enzymes involved in steroidogenesis, thus playing a crucial role in the nutritional regulation of steroid production responsible for sustained steroidogenesis over an extended period. The intricate link between male testosterone and lipid metabolism is evident. Lee et al. observed a negative correlation between testosterone and one-tenth of triglycerides, a positive correlation between testosterone and one-tenth of high-density lipoprotein cholesterol, and an inverted U-shaped correlation between testosterone and one-tenth of low-density lipoprotein (8). In a cross-sectional study conducted by Liu et al., elevated lipid accumulation products were found to be associated with a higher incidence of testosterone loss and deficiency, particularly in individuals with hypertension and non-smokers (9). Lipid accumulation products have a certain significance in predicting testosterone deficiency (9). A study by Hurley demonstrated that the use of high-dose androgens resulted in a 50% reduction in HDL-C and an increase of over 50% in LDL-C (10). Furthermore, the biosynthesis and secretion of testosterone exhibit a tight correlation with age. During puberty, testosterone levels peak in response to luteinizing hormone stimulation and subsequently decline with advancing age (11). A study conducted by Mohr BA et al. (12) established percentile thresholds for testosterone level fluctuations in distinct age cohorts, thereby revealing substantial horizontal and vertical associations between serum TT, CFT, bioactive testosterone levels, and age. Additionally, several observational studies suggest an association between low testosterone levels in males and adverse cardiovascular outcomes (13, 14).

Recent evidence suggests that while lipid-lowering therapies targeting serum LDL-C levels reduce the risk of atherosclerotic cardiovascular disease (ASCVD) in the general population, some individuals with normal or low LDL-C concentrations still experience ASCVD-related events, and some may even show the progression of atherosclerosis (15). Some researchers have constructed Cox proportional hazard models to analyze data, revealing that for each increase of one standard unit in non-HDL-C cholesterol and apoB levels, there is a 19% escalation in the incidence rate of major cardiovascular events, surpassing the impact of LDL-C levels (15%). The predictive capability of apoB (HR = 1.24) and non-HDL-C cholesterol (HR = 1.31) was more pronounced than that of LDL-C. Subsequent research further indicated that in assessing residual cardiovascular risk following statin therapy, apoB, and non-HDL-C cholesterol levels exhibited higher predictive value (16). A meta-analysis by Sniderman et al. (17) in 2011 found that compared to a 40% reduction in LDL-C levels, a 40% reduction in non-HDL-C cholesterol would reduce cardiovascular events by 200,000 over ten years, while a 40% reduction in apoB would prevent 500,000 cardiovascular events. This highlights the superior predictive value of apoB compared to LDL-C and non-HDL-C in assessing cardiovascular benefits of lipid-lowering therapy, further endorsing apoB as a crucial treatment target, especially post-achievement of LDL-C targets.

Exploring the relationship between testosterone and apoB dates back to Stefanick’s study in 1987 (18). In our study, we investigated the relationship between testosterone and apoB in diverse populations and exposure factors. Among individuals without cancer, we explored the linear relationship between serum TT and serum apoB in various CVD, and visually depicted the relationship using a smooth curve fitting plot. Investigating the correlation between serum apoB and serum TT offers valuable insights into screening individuals with potential CVD within the population exhibiting normal or low LDL-C levels. Notably, certain guidelines currently propose apoB as a secondary target for intervention in blood lipid management, aiming to reduce the residual risk of ASCVD in patients (19, 20). Therefore, exploring the association between apoB and TT contributes significantly to the comprehensive understanding and management of CVD.

## Methods

### Study design and population

The NHANES is an extensive health and nutrition survey conducted by the National Center for Health Statistics in the United States. Since its initiation in the early 1960s, NHANES has been dedicated to evaluating the health and nutritional status of individuals throughout the country. By conducting family interviews and physical examinations, this survey gathers comprehensive information encompassing biological, social, psychological, behavioral, and demographic aspects, all provided at no cost to participants. In this cross-sectional study, we utilized NHANES data from 2011 to 2016 to explore the relationship between apoB and total TT within the general population.

### Sample selection

We conducted our analysis using the NHANES database, which comprised a total of 29,902 participants from the years 2011 to 2016. The dataset encompassed various demographic variables, including age, race, ratio of family income to poverty, marital status, and education level. Additionally, we considered several cardiovascular disease risk factors such as hypertension, high cholesterol levels, diabetes, BMI, drinking habits, and smoking variables. It also includes Vigorous work activity. All data can be found at the following URL: www.cdc.gov/nchs/nhanes/ (Date of access online: January 12, 2024).

**Figure 1:**
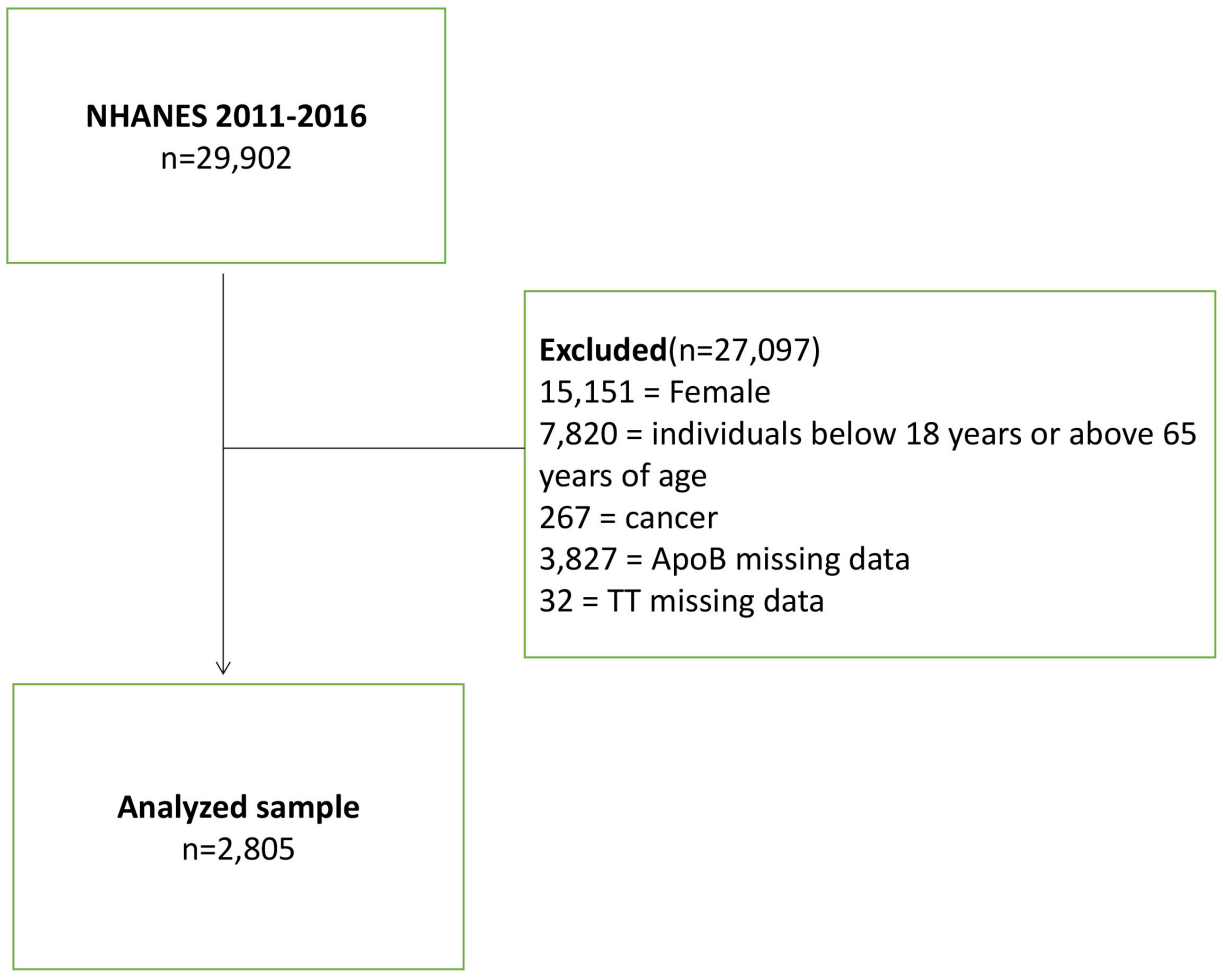
Flow chart of the study population.

From the initial pool of 29,902 participants, we excluded those who were female (n=15,151), individuals below 18 years or above 65 years of age (n=7,820), participants with a cancer diagnosis (n=267), and those with missing data for apoB (n=3,827) and total testosterone (n=32). Consequently, a total of 2,805 participants were included in this cross-sectional study (**Figure 1**). It is important to note that all NHANES research participants from 2011 to 2016 provided informed consent, and the study protocol obtained approval from the Research Ethics Review Committee of the National Health Statistics Center.

### Variables

The demographic variables considered in this study were age, race (including Mexican American, other Hispanic, non-Hispanic white, non-Hispanic black, or other race), the ratio of family income to poverty, marital status, and education level. For this study, the age range of 18 to 65 years was chosen due to the known variations in testosterone levels across different age groups. Testosterone levels are typically low in children under 10 years of age, start to increase during the ages of 10 to 15 with the onset of puberty (21), and reach their peak around the age of 19 (22). However, as men continue to age, testosterone levels gradually decline, particularly in older men above the age of 65 (23). To classify the education level variable, individuals with educational attainment below the 9th grade, those with 9th-11th grade education (including 12th grade without a diploma), and high school graduates or equivalent were categorized as the “High School Grad and Less Than” group. On the other hand, individuals with some college or associate degrees, as well as college graduates or above, were classified as the “Above” group. Thus, the education level variable was dichotomized as “High School Grad and Less Than” versus “Above.”

The questionnaire variables in this study encompassed several factors. These included hypertension (categorized as “No” or “Yes”), high cholesterol levels (“No” or “Yes”), diabetes (“No” or “Yes”), drinking (“No” or “Yes”), smoke (“Not at all,” “Every day,” or “Some days”), and vigorous work activity (“No” or “Yes”). To define alcohol users, individuals who consumed a minimum of 12 drinks within the past 12 months were considered (24). Therefore, we used the threshold of 12 drinks in the past 12 months as the criterion to categorize the drinking variable into a binary form, namely “Drinking” (“No” or “Yes”).

The examination variable included body mass index (BMI), which was treated as a continuous variable. For analysis, BMI was categorized into two groups: < 25 kg/m² and ≥ 25 kg/m².

For the NHANES laboratory methodology regarding the determination of TT, further information can be accessed at: https://wwwn.cdc.gov/Nchs/Nhanes/2011-2012/TST_G.htm (Date of access online: January 12, 2024). Similarly, detailed information regarding the NHANES laboratory methodology for apoB determination is available at: https://wwwn.cdc.gov/Nchs/Nhanes/2011-2012/APOB_G.htm (Date of access online: January 12, 2024). Additional covariates can be found at the following URL: www.cdc.gov/nchs/nhanes/ (Date of access online: January 12, 2024).

### Data analysis

Given the intricate probability cluster design of NHANES, individual sample weights were assigned to each survey participant, and all statistical analyses in this study took into account these weights. We conducted statistical analyses in accordance with the guidelines outlined by the Centers for Disease Control (CDC) (25). To study the associations of participant characteristics, we utilized weighted linear regression and the Chi-square test where appropriate. Initially, we conducted a weighted univariate analysis to examine the relationship between TT and the covariates. Subsequently, we conducted weighted multifactor analyses while adjusting for covariates such as age, race, education level, ratio of family income to poverty, marital status, hypertension, high cholesterol levels, BMI, diabetes, drinking, smoking, and vigorous work activity. Furthermore, to confirm the relationship between apoB and TT, we performed smooth curve fitting using apoB (g/L) as the independent variable and TT (ng/dL) levels as the dependent variable. This analysis allowed us to visualize and assess the relationship between apoB and TT.

All statistical analyses were carried out using Empowerstats (https://www.empowerstats.net/cn/) and R software. All estimates were weighted using appropriate NHANES sample weights. Following guidelines from the Centers for Disease Control and Prevention, weighted models were used to address oversampling of minority ethnicities, ensuring fair and accurate estimates of the population impact. Results were considered statistically significant when p-values were less than 0.05.

## Results

**Table 1** presents 14 study variables, including the independent variable ApoB, the dependent variable TT, and covariates Age, Ratio of family income to poverty, Race, Education level, Marital status, Diabetes, Smoking, Drinking, Hypertension, High cholesterol level, Vigorous work activity, and BMI. The analysis sample comprises 2,805 male participants. We divided ApoB into quartiles Q1-Q4, corresponding to mean ages of 36.3, 40.9, 43.1, and 43.9 years, and corresponding TT levels of 501.1, 472.1, 442.7, and 427.1 ng/dL, respectively. For continuous variables, we calculated weighted means (95% CI) and determined P-values using survey-weighted linear regression (svyglm). For categorical variables, we calculated weighted percentages (95% CI) and obtained P-values using survey-weighted chi-square tests (svytable).

**Table 1:** Characteristics of participants, Weighted (N =2,805).

ApoB (g/L) quartile	Q1 <0.7g/L	Q2 ≥0.7g/L to <0.9g/L	Q3 ≥0.9g/L to <1.1g/L	Q4 ≥1.1g/L	P-value
N	691	710	696	708	
ApoB (g/L)	0.6 (0.6,0.6)	0.8 (0.8,0.8)	1.0 (1.0,1.0)	1.3 (1.3,1.3)	<0.001
TT (ng/dL)	501.1 (481.6,520.5)	472.1 (454.9,489.4)	442.7 (424.6,460.8)	427.1 (411.7,442.4)	<0.001
Age (years)	36.3 (34.8,37.7)	40.9 (39.4,42.4)	43.1 (41.9,44.3)	43.9 (42.8,45.0)	<0.001
Ratio of family income to poverty	2.7 (2.5,3.0)	2.9 (2.7,3.2)	3.1 (2.9,3.3)	2.8 (2.6,3.0)	0.010
Race					<0.001
Mexican American	8.8 (6.3,12.2)	9.1 (6.2,13.3)	11.1 (8.0,15.2)	13.5 (10.1,17.8)	
Other Hispanic	6.0 (4.2,8.5)	5.9 (4.0,8.5)	7.6 (5.5,10.3)	9.4 (6.6,13.2)	
Non-Hispanic White	63.8 (57.3,69.8)	65.4 (59.2,71.1)	65.0 (58.8,70.8)	60.8 (54.1,67.0)	
Non-Hispanic Black	13.2 (9.9,17.4)	9.9 (7.7,12.7)	7.4 (5.8,9.4)	8.2 (6.2,10.8)	
Other Race	8.3 (6.7,10.2)	9.8 (7.6,12.5)	8.9 (6.7,11.6)	8.1 (6.2,10.7)	
Education level					0.058
High School Grad and Less Than	36.9 (31.6,42.6)	37.6 (32.0,43.5)	39.0 (32.1,46.4)	46.7 (39.9,53.6)	
Above	63.1 (57.4,68.4)	62.4 (56.5,68.0)	61.0 (53.6,67.9)	53.3 (46.4,60.1)	
Marital status					<0.001
married or with partners	53.3 (48.0,58.5)	63.7 (57.5,69.4)	69.2 (65.0,73.2)	68.1 (63.0,72.8)	
widowed or divorced	9.0 (6.5,12.3)	10.8 (7.7,15.0)	9.1 (6.5,12.7)	12.5 (10.0,15.4)	
unmarried	36.5 (31.9,41.4)	23.8 (19.5,28.7)	20.1 (16.7,24.1)	16.8 (13.1,21.2)	
separated	1.2 (0.6,2.2)	1.7 (1.1,2.6)	1.5 (0.7,3.4)	2.7 (1.7,4.3)	
Diabetes					0.686
No	90.8 (87.4,93.4)	92.2 (89.4,94.4)	92.9 (89.4,95.4)	92.7 (90.3,94.5)	
Yes	9.2 (6.6,12.6)	7.8 (5.6,10.6)	7.1 (4.6,10.6)	7.3 (5.5,9.7)	
Smoke					0.402
Not at all	45.3 (36.8,54.2)	54.9 (47.3,62.2)	54.4 (46.8,61.8)	48.0 (41.0,55.1)	
Every day	43.6 (35.9,51.7)	35.0 (28.3,42.4)	33.3 (26.9,40.4)	40.5 (33.0,48.4)	
Some days	11.0 (7.0,16.8)	10.1 (7.0,14.5)	12.3 (8.4,17.8)	11.5 (7.3,17.7)	
Drinking					0.653
No	96.9 (93.2,98.6)	97.7 (95.8,98.8)	97.1 (94.4,98.6)	98.3 (96.9,99.1)	
Yes	3.1 (1.4,6.8)	2.3 (1.2,4.2)	2.9 (1.4,5.6)	1.7 (0.9,3.1)	
Hypertension					0.007
No	78.5 (74.9,81.8)	73.1 (67.5,78.1)	73.0 (68.9,76.7)	67.1 (61.2,72.5)	
Yes	21.5 (18.2,25.1)	26.9 (21.9,32.5)	27.0 (23.3,31.1)	32.9 (27.5,38.8)	
High cholesterol level					<0.001
No	81.6 (76.6,85.7)	76.0 (71.3,80.1)	66.5 (61.4,71.3)	55.9 (50.6,61.0)	
Yes	18.4 (14.3,23.4)	24.0 (19.9,28.7)	33.5 (28.7,38.6)	44.1 (39.0,49.4)	
Vigorous work activity					0.298
No	68.3 (63.2,73.1)	69.2 (65.7,72.4)	70.7 (65.1,75.7)	64.5 (58.1,70.4)	
Yes	31.7 (26.9,36.8)	30.8 (27.6,34.3)	29.3 (24.3,34.9)	35.5 (29.6,41.9)	
BMI (kg/m2)					<0.001
<25	45.6 (40.3,50.9)	30.1 (25.9,34.7)	22.0 (18.1,26.4)	16.2 (13.0,20.1)	
>=25	54.4 (49.1,59.7)	69.9 (65.3,74.1)	78.0 (73.6,81.9)	83.8 (79.9,87.0)	

Data in **Table 1**:

For continuous variables: survey-weighted mean (95% CI), P-value was by survey-weighted linear regression.

For categorical variables: survey-weighted percentage (95% CI), P-value was by survey-weighted Chi-square test.

**Table 2** presents the univariate analysis results for 13 variables related to the dependent variable TT. The table reveals a close and statistically significant association between serum ApoB and TT. Additionally, Ratio of family income to poverty, Age, Marital status, Hypertension, High cholesterol level, Diabetes, Smoking, and BMI are also linked to TT. For each unit increase in Ratio of family income to poverty, TT decreases by 6.2 ng/dL. With every year’s increment in Age, TT decreases by 1.2 ng/dL. Regarding Marital status, the “unmarried” group exhibits a 53.3 ng/dL increase in TT compared to the “married or with partners” group. Hypertension is associated with a 56.7 ng/dL decrease in TT compared to individuals without Hypertension. High cholesterol level is linked to a TT reduction of 42.5 ng/dL compared to those without High cholesterol level. Each 1 kg/m² increase in BMI corresponds to a 13.1 ng/dL decrease in TT. When dichotomized, individuals with a BMI ≥25 kg/m² experience a 151.6 ng/dL decrease in TT compared to those with a BMI <25 kg/m². Diabetes individuals witness a TT decrease of 72.5 ng/dL compared to non-Diabetes individuals. In the Smoking category, compared to “Not at all,” the “Every day” and “Some days” groups show TT increases of 46.2 ng/dL and 51.3 ng/dL, respectively.

**Table 2:** Univariate analysis for TT, Weighted.

Covariate	Mean/percentage (95% CI)	β (95%CI)	P-value
ApoB (g/L)	1.0 (0.9,1.0)	-113.4 (-146.6, -80.2)	<0.001
ApoB (g/L) quartile			
Q1	23.3 (21.2,25.6)	Ref	
Q2	26.1 (24.2,28.0)	-28.9 (-50.9, -7.0)	0.013
Q3	25.8 (23.2,28.6)	-58.4 (-85.8, -30.9)	<0.001
Q4	24.8 (22.2,27.6)	-74.0 (-97.3, 50.7)	<0.001
Ratio of family income to poverty	2.9 (2.8,3.0)	-6.2 (-11.7, -0.76)	0.031
Ratio of family income to poverty Tertile			
Low	23.0 (20.1,26.2)	Ref	
Middle	32.3 (29.9,34.8)	-24.8 (-43.0, -6.5)	0.010
High	44.7 (40.7,48.8)	-29.1(-52.0, -6.30)	0.016
Age(years)	41.1 (40.4,41.9)	-1.2 (-2.1, -0.35)	0.009
Age (years) Tertile			
Low	31.5 (28.9,34.2)	Ref	
Middle	35.1 (32.4,37.8)	-63.1 (-81.7, -44.4)	<0.001
High	33.4 (30.9,36.1)	-43.1 (-73.2, -13.0)	0.007
Race			
Mexican American	10.6 (8.2,13.6)	Ref	
Other Hispanic	7.2 (5.5,9.4)	-17.0 (-61.3, 27.3)	0.456
Non-Hispanic White	63.8 (58.8,68.5)	-9.6 (-32.9, 13.7)	0.424
Non-Hispanic Black	9.6 (7.7,11.9)	17.3 (-13.3, 47.8)	0.274
Other Race	8.8 (7.3,10.5)	1.4 (-25.2, 28.1)	0.916
Education level			
High School Grad and Less Than	40.2 (36.2,44.3)	Ref	
Above	59.8 (55.7,63.8)	-14.0 (-34.0, 6.0)	0.177
Marital status			
married or with partners	64.0 (61.1,66.9)	Ref	
widowed or divorced	10.4 (8.9,12.1)	-2.5 (-25.8, 20.8)	0.833
unmarried	23.8 (21.4,26.4)	53.3 (28.8, 77.8)	<0.001
separated	1.8 (1.3,2.5)	42.4 (-26.0, 110.9)	0.230
Hypertension			
No	72.9 (70.3,75.3)	Ref	
Yes	27.1 (24.7,29.7)	-56.7 (-78.9, -34.5)	<0.001
High cholesterol level			
No	69.8 (67.1,72.3)	Ref	
Yes	30.2 (27.7,32.9)	-42.5 (-57.8, -27.2)	<0.001
BMI(kg/m2)	28.8 (28.4,29.2)	-13.1 (-14.2, -11.9)	<0.001
BMI (kg/m2) dichotomous			
<25	28.2 (26.0,30.5)	Ref	
>=25	71.8 (69.5,74.0)	-151.6 (-171.6, -131.6)	<0.001
Diabetes			
No	92.2 (90.7,93.4)	Ref	
Yes	7.8 (6.6,9.3)	-72.5 (-116.1, -28.9)	0.002
Drinking			
No	97.5 (96.5,98.3)	Ref	
Yes	2.5 (1.7,3.5)	5.3 (-46.2, 56.9)	0.840
Smoke			
Not at all	50.9 (46.7,55.2)	Ref	
Every day	37.8 (33.4,42.4)	46.2 (14.0, 78.3)	0.007
Some days	11.3 (9.1,13.9)	51.3 (5.3, 97.2)	0.034
Vigorous work activity			
No	68.2 (65.3,71.0)	Ref	
Yes	31.8 (29.0,34.7)	7.8 (-9.6, 25.3)	0.384

**Table 3** presents the model results, indicating that in the unadjusted model, there is a negative correlation between apoB concentration and serum TT concentration (β=-113.4; 95% CI: -146.6, -80.2; P<0.001). This implies that for every 1g/L increase in apoB, serum TT decreases by 113.4ng/dL, with statistical significance. In the adjusted model (β=-61.0; 95% CI: -116.7, -5.2; P=0.040), each 1g/L increase in apoB is associated with a 61.0ng/dL decrease in serum TT, and this association is statistically significant. The conversion of the apoB concentration from a continuous variable to a categorical variable (quartile: Q1<0.7g/L; Q2≥0.7g/L to <0.9g/L; Q3≥0.9g/L to <1.1g/L; Q4≥1.1g/L) revealed that the level of TT of the participants in the highest quartile (≥1.1g/L) was -47.2 pg/mL (95% CI: -91.2, -3.3; P=0.045) lower than that in the lowest quartile (<0.7g/L).

**Table 3:** Relationship between ApoB and TT, Weighted.

Outcome	Crude Model		Adjusted Model	
	βor OR (95%CI)	P-value	βor OR (95%CI)	P-value
ApoB (g/L)	-113.4 (-146.6, -80.2)	<0.001	-61.0 (-116.7, -5.2)	0.040
ApoB (g/L) quartile				
Q1 <0.7g/L	Ref		Ref	
Q2 ≥0.7g/L to <0.9g/L	-28.9 (-50.9, -7.0)	0.013	-26.7 (-61.8, 8.4)	0.148
Q3 ≥0.9g/L to <1.1g/L	-58.4 (-85.8, -30.9)	<0.001	-12.5 (-59.7, 34.6)	0.606
Q4 ≥1.1g/L	-74.0 (-97.3, 50.7)	<0.001	-47.2 (-91.2, -3.3)	0.045

Data in the table: β or OR (95%CI); P-value.

Result variable: TT (ng/dL).

Exposure variable: ApoB(g/L); ApoB(g/L) quartile.

The adjusted model adjusts for Age; Race (Mexican American, Other Hispanic, Non-Hispanic White, Non-Hispanic Black, Other Race); Education level (High School Grad and Less Than and Above); Ratio of family income to poverty; Marital status (married or with partners, widowed or divorced, unmarried, and separated); Hypertension (No, Yes); High cholesterol level (No, Yes); BMI (<=25, >25); Diabetes (No, Yes); Drinking (No, Yes); Smoke (Not at all, Every day, and Some days); Vigorous work activity (No, Yes).

It can be seen that after adjusting the relevant variables, apoB was linearly correlated with TT, and TT decreased with the increase of apoB.

**Figure 2:**
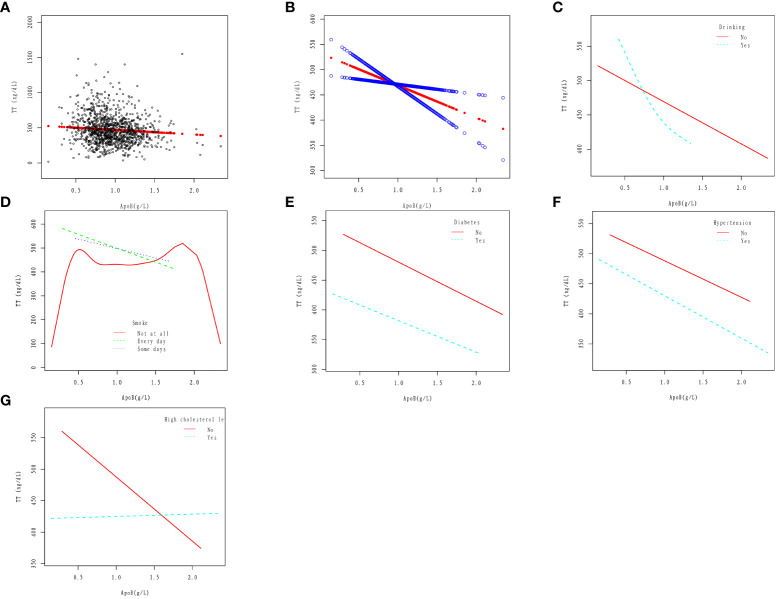
Association between apoB and TT. **(A)** Cross-combination modeling of each apoB and TT. A black dot represents a sample. **(B)** A linear association between apoB and TT was found in a generalized additive model (GAM). The Solid red line represents the smooth curve fit between variables. Blue bands represent the 95% confidence interval from the fit. The results were obtained after adjusting for Age, Race, Education level, Ratio of family income to poverty, Marital status, Hypertension, High cholesterol level, BMI, Diabetes, Drinking, Smoke, and Vigorous work activity. **(C)** Association between apoB and serum Klotho in Drinking (No, Yes) group. **(D)** Association between apoB and serum TT in Smoke (No, Yes) group. **(E)** Association between apoB and serum TT in Diabetes (No, Yes) group. **(F)** Association between apoB and serum TT in Hypertension (No, Yes) group. **(G)** Association between apoB and serum TT in High cholesterol level (No, Yes) group.

In the drinking population, TT showed a downward trend with increasing apoB, and then the downward trend tended to slow down. In the non-alcohol drinkers, apoB was linearly correlated with TT, and TT decreased with the increase of apoB. The results were obtained after adjusting for Age, Race, Education level, Ratio of family income to poverty, Marital status, Hypertension, High cholesterol level, BMI, Diabetes, Smoke, and Vigorous work activity (**Figure 2C**).

In the smoker(Every day and Some days smoke)population, apoB was linearly correlated with TT, and TT decreased with the increase of apoB. In the non-smoker population, The relationship between apoB and TT loses its linear correlation. The results were obtained after adjusting for Age, Race, Education level, Ratio of family income to poverty, Marital status, Hypertension, High cholesterol level, BMI, Diabetes, Drinking, and Vigorous work activity (**Figure 2D**).

In diabetic and non-diabetic patients, apoB was linearly correlated with TT, and TT decreased with the increase of apoB. The results were obtained after adjusting for Age, Race, Education level, Ratio of family income to poverty, Marital status, Hypertension, High cholesterol level, BMI, Drinking, Smoke, and Vigorous work activity (**Figure 2E**).

In the hypertensive and non-hypertensive population, apoB was linearly correlated with TT, and TT decreased with the increase of apoB. The results were obtained after adjusting for Age, Race, Education level, Ratio of family income to poverty, Marital status, High cholesterol level, BMI, Diabetes, Drinking, Smoke, and Vigorous work activity (**Figure 2F**).

In the high cholesterol level population, the change in TT is not significant with the increase of apoB; In the non-high cholesterol level population, apoB was linearly correlated with TT, and TT decreased with the increase of apoB. The results were obtained after adjusting for Age, Race, Education level, Ratio of family income to poverty, Marital status, Hypertension, BMI, Diabetes, Drinking, Smoke, and Vigorous work activity (**Figure 2G**).

## Discussion

According to the findings of this study, in the population of adult males with an average age of 41.1 years and without cancer, there exists a negative linear correlation between serum apoB and TT, as illustrated in **Figures 2A, B**. This correlation remains consistent even in the presence of several cardiovascular risk factors such as smoking, diabetes, and hypertension, as depicted in **Figures 2D-F**. However, the relationship between apoB and TT takes a different pattern among individuals with alcohol consumption and high cholesterol levels. In the population with alcohol consumption, TT shows a declining trend as apoB increases, followed by a slower rate of decline (**Figure 2C**). On the other hand, in the population with high cholesterol levels, the change in TT is not significantly evident with increasing apoB (**Figure 2G**). Based on two crucial theoretical concepts: the strong correlation between apoB and LDL-C and the role of apoB as a potential risk factor for CVD (26). The association between testosterone and CVD is easily conceivable. Some researchers have pointed out that testosterone and its synthetic derivatives are related to sudden cardiac death (27, 28). As demonstrated by epidemiological research, individuals who misuse steroids are more prone to engage in criminal activities, with steroids occasionally identified as an indirect cause of death (29, 30). Kintz et al., based on the increased risk of antisocial lifestyle associated with steroid hormones, explored methods for detecting steroid hormones through body hair analysis (31). Thus, elevated levels of apoB may serve as a significant predictor of CVD risk, particularly in individuals with normal or low LDL-C levels. Consequently, further investigation into the relationship between apoB and TT could prove valuable in identifying potential CVD patients within this population. Additionally, for the treatment of testosterone deficiency, apart from direct testosterone supplementation, maintaining apoB at a certain level may also hold promise as a potential adjunctive treatment.

Several studies have explored the potential connection between serum TT and blood lipids. These studies have revealed that testosterone deficiency can lead to elevated serum TG levels and increased white adipose tissue in male mice fed a high-fat diet (32). Additionally, the San Antonio Heart Research Center found negative correlations between testosterone and TG, TC, and LDL-C, and a positive correlation with HDL-C (33). Similar associations were observed in the Tromsa study, with testosterone being negatively correlated with TG and positively correlated with HDL-C (34). The Turku Male Aging Study reported positive correlations between testosterone and HDL-C, and negative correlations with TC and TG, although no statistically significant relationship was observed with LDL-C (35). These findings demonstrate the close relationship between testosterone and CVD-related factors such as HDL-C, LDL-C, TG, and TC. However, the investigation of the relationship between apoB and serum TT has received limited attention thus far. Previous evidence has demonstrated the beneficial effects of testosterone replacement therapy in male patients with hypogonadism, including improvements in certain cardiovascular risk factors (36). Elevated serum testosterone levels can lead to adverse outcomes. Coward et al. found that the administration of synthetic androgens may result in testicular dysfunction. Males under 50 years old, previously exposed to assimilative androgens, exhibit over 10 times the likelihood of testicular dysfunction compared to those aged 50 and above, and these findings are statistically significant (37). In a rat model, Shirpoor et al. observed cardiac and aortic remodeling induced by the synthetic androgenic steroid, Nandrolone. They documented an increase in systolic pressure, diastolic pressure, mean arterial pressure, and necrotic pressure (38). Esposito et al. summarized that the misuse of synthetic androgenic steroids can induce various side effects on organ systems, including the reproductive system (39).

In our study, we examined the association between serum apoB and TT, while considering various potential confounders including age, race, education level, ratio of family income to poverty, marital status, hypertension, high cholesterol level, BMI, diabetes, drinking, smoking, and vigorous work activity. Through the use of linear regression models and smooth curve fitting, we discovered a negative correlation between serum apoB concentration and TT concentration. Overall, our study sheds light on an underexplored area of research and provides valuable insights into the association between serum apoB and TT in the context of cardiovascular health in adult males without cancer. These findings contribute to our understanding of the complex interplay between lipoproteins, testosterone, and cardiovascular risk factors.

Lee et al. utilized the Framingham Risk Score (FRS) to assess the impact of testosterone on cardiovascular disease in patients with sexual dysfunction (40). The findings revealed an association between testosterone levels and FRS in the sexual dysfunction cohort, suggesting that elevated testosterone levels may reduce the risk of cardiovascular disease in these individuals. In another study, Grandys et al. found that reduced serum testosterone concentrations were associated with increased inflammation and worsening of blood lipids in men (41). Diverging from prior research (18, 40, 41), our study investigates the association between serum TT and apoB across diverse populations and exposure factors. Within individuals devoid of cancer, we explore the linear relationship between serum TT and apoB in various CVD. This study represents a pioneering effort to investigate the correlation between serum TT and the potential cardiovascular risk factor apoB using publicly available NHANES data. While this study offers notable strengths, it is crucial to recognize and address its limitations. Firstly, the cross-sectional design of the NHANES database restricts our ability to establish any causal relationship between human serum apoB and TT. Secondly, it is plausible that there are unidentified confounding variables that could influence serum TT and apoB levels, which were not accounted for in this study. It is important to consider these limitations when interpreting the findings and to encourage further research to explore these associations in more depth.

## Conclusions

This nationally representative study indicates a negative linear correlation between serum ApoB and TT in adult American males without cancer. Upon adjustment, for every 1 g/L increase in ApoB, there is a significant decrease of 61.0 ng/dL in TT. This suggests that reducing ApoB concentration could be beneficial in preventing low testosterone occurrence. Furthermore, low testosterone levels can serve as predictive indicators for the development of CVD. These findings offer a promising avenue for the prevention and treatment of both low testosterone and cardiovascular disease. In general, investigating the relationship between apoB and serum testosterone levels can yield profound insights into preventing and managing testosterone deficiency, aiding in the development of more effective preventive and therapeutic strategies. This necessitates a comprehensive consideration of multiple factors, including lipid metabolism, cardiovascular health, and lifestyle factors.

## Data availability statement

The datasets presented in this study can be found in online repositories. The names of the repository/repositories and accession number(s) can be found below: www.cdc.gov/nchs/nhanes/.

## Ethics statement

The study was conducted by following the Declaration of Helsinki, and the National Center for Health Statistics institutional review board approved the overall NHANES. This study was approved by the Institutional Review Board and documented consent was obtained from participants. The studies were conducted in accordance with the local legislation and institutional requirements. The participants provided their written informed consent to participate in this study. Written informed consent was obtained from the individual(s) for the publication of any potentially identifiable images or data included in this article.

## Author contributions

ZC: Conceptualization, Data curation, Formal analysis, Investigation, Methodology, Software, Writing – original draft, Writing – review & editing. EZ: Writing – original draft. LG: Writing – original draft. GJ: Writing – original draft. QD: Writing – original draft. MH: Conceptualization, Writing – original draft. HL: Conceptualization, Writing – original draft. GH: Conceptualization, Funding acquisition, Supervision, Writing – original draft.
